# Beta synchrony for expressive language lateralizes to right hemisphere in development

**DOI:** 10.1038/s41598-021-83373-z

**Published:** 2021-02-17

**Authors:** Vivek V. Sharma, Jennifer Vannest, Hansel M. Greiner, Hisako Fujiwara, Jeffrey R. Tenney, Brady J. Williamson, Darren S. Kadis

**Affiliations:** 1 Neurosciences and Mental Health, Hospital for Sick Children, 686 Bay Street, Toronto, ON M5G 0A4 Canada; 2 Communication Sciences & Disorders, University of Cincinnati, Cincinnati, OH USA; 3 Division of Speech-Language Pathology, Cincinnati Children’s Hospital Medical Center, Cincinnati, OH USA; 4 Division of Neurology, Cincinnati Children’s Hospital Medical Center, Cincinnati, OH USA; 5 Department of Radiology, University of Cincinnati, Cincinnati, OH USA; 6 Department of Physiology, Faculty of Medicine, University of Toronto, Toronto, ON Canada

**Keywords:** Language, Neuroscience

## Abstract

A left perisylvian network is known to support language in healthy adults. Low-beta (13–23 Hz) event-related desynchrony (ERD) has been observed during verb generation, at approximately 700–1200 ms post-stimulus presentation in past studies; the signal is known to reflect increased neuronal firing and metabolic demand during language production. In contrast, concurrent beta event-related synchrony (ERS) is thought to reflect neuronal inhibition but has not been well studied in the context of language. Further, while low-beta ERD for expressive language has been found to gradually shift from bilateral in childhood to left hemispheric by early adulthood, developmental lateralization of ERS has not been established. We used magnetoencephalography to study low beta ERS lateralization in a group of children and adolescents (*n* = 78), aged 4 to less than 19 years, who performed covert verb generation. We found that the youngest children had bilateral ERD and ERS. By adolescence, low-beta ERD was predominantly left lateralized in perisylvian cortex (i.e., Broca’s and Wernicke’s regions), while beta ERS was predominantly right lateralized. Increasing lateralization was significantly correlated to age for both ERD (Spearman’s *r* = 0.45, *p* < 0.01) and ERS (Spearman’s *r* =  − 0.44, *p* < 0.01). Interestingly, while ERD lateralized in a linear manner, ERS lateralization followed a nonlinear trajectory, suggesting distinct developmental trajectories. Implications to early-age neuroplasticity and neuronal inhibition are discussed.

## Introduction

Left perisylvian cortex, including Broca’s and Wernicke’s areas, is known to support gross language processes in healthy adolescents and adults^1–13^. The network becomes increasingly left-lateralized and focal by adolescence^14,15^, unless development is disrupted by early neurological insult^16–19^. Since children show greater propensity for recruiting extracanonical regions to support language, understanding the timing for normal developmental shifts in representation may inform clinical practice.

Event-related desynchronization (ERD) and event-related synchronization (ERS) reflect band-limited power changes in neuronal oscillations. ERD reflects decreases in spectral power relative to a baseline, while ERS reflects increases. ERD and ERS occur either at the same time point in different spatial locations, or in the same location at different moments of time^20^. Originally, increased band-limited alpha (8–12 Hz) and beta (13–29 Hz) signals were described as idle rhythms, which is to say that alpha and beta ERS was observed during wakeful rest. More recent studies have found alpha/beta ERD at spatial locations and time points thought to be critically involved in tasks, unlike ERS, which was found contralaterally^21–23^. These studies associated alpha/beta ERD with the task-related removal of neuronal inhibition and ERS with increases in inhibition. This represents a shift from early cortical idling theory; the role of ERS in functional inhibition is now well-established^24,25^.

Beta ERD is correlated with the blood oxygen level dependent (BOLD) signal in fMRI, including for language^26–28^. ERD has been found during phonetic processing in typically developing children and adolescents, but not in children with developmental language impairments such as aphasia and dyslexia^29^. Indeed, low-beta (13–23 Hz) ERD is considered an oscillatory signature for expressive language in both children and adults^30^. Studies have found this ERD at perisylvian language networks as early as 650 ms and as late as 1200 ms post-stimulus presentation, which is thought to be locked to response timing^31–33^.

While ERD as a signature of expressive language is now established in terms of temporal dynamics and spatial localization, the role of ERS has not been characterized for language. Here, we lateralize and localize low-beta ERS for language in development using a covert auditory verb generation task in MEG. Beta ERS is thought to represent active inhibition in interneuron functioning—as such, we hypothesize that ERS lateralization emerges as part of the normal developmental trajectory of inhibitory function, and may index age-related plasticity of cortical language representation, which has implications on accommodation of lesions^34,35^. Thus, ERS may complement ERD for indexing language lateralization, and perhaps assay plastic potential, in development.

## Method

### Participants

Eighty-two typically developing children, ages 4.0 to less than 19.0 years, participated in this study (Fig. S1). All participants were native English speakers, without history of neurological insult, speech or language impairment, or learning disability. Handedness was determined by the Edinburgh Handedness Inventory (EHI)^36^. Informed written consent from a parent and/or legal guardian was obtained for all participants under the age of 18.0 years; children ages 10.0 to 18.0 provided assent, prior to participation. Participants 18.0 to less than 19 years provided informed written consent, directly. The study was approved by the Institutional Review Board at Cincinnati Children’s Hospital Medical Center and conforms to the guidelines and regulations of the US Federal Policy for the Protection of Human Subjects. Participants received compensation for their participation and travel.

### Verb generation paradigm

Participants heard either a concrete noun (verb generation trials), or speech-shaped noise (control trials). Participants were instructed to rapidly think of an action word that corresponded to each target noun during the verb generation trials. Nouns consisted of everyday items chosen from normative databases and standardized language assessments; items are familiar to a typically developing 5-year-old (e.g., pencil, book, dog, etc.). Each verb generation trial requires auditory comprehension, semantic processing (i.e., associating the noun with appropriate verbs) and covert language production (i.e., verb generation). During the control trials, participants were asked to listen to noise stimuli, without responding. This paradigm has been described previously^37^. Noun and noise stimuli were presented randomly, with an inter-stimulus interval jittered between 4 and 5 s. A total of 71 distinct nouns and 71 noise trials were presented. Noun and noise stimuli were identical in duration (670 ms ± 140 ms). The participants were trained on an overt version of this paradigm prior to task and MEG recording, which established sufficient verb generation ability (at least 8/10 items correct) and promoted task compliance during subsequent data acquisition. Following acquisition, participants were asked to overtly recall nouns and corresponding verbs that were presented; in all cases, participants were able to recall several items.

Neuropsychological assessments included the Peabody Picture Vocabular Test (PPVT-IV) and Expressive Vocabulary Test (EVT-II) in 26 participants, and the Clinical Evaluation of Language Fundamentals (CELF 4th Edition) in 56 participants^37–39^. Scores from the PPVT and EVT were averaged as an approximation for verbal intelligence; likewise, the CELF Total score was used to assay verbal intelligence. These measures have a population mean of 100, and a standard deviation of 15.

### Data acquisition

#### MEG data acquisition

MEG data were acquired using a whole-head 275-channel CTF system (MEG International Services Ltd., Coquitlam, B.C., Canada) with a sampling rate of 1.2 kHz. Participants were tested in the supine position with the heads supported on memory foam and/or linens for stability and comfort. MEG sessions were limited to less than 27 min of data collection, in order to minimize fatigue and maximize participation in the studies. Stimuli were presented binaurally by an intensity calibrated distal transducer through tubing and ear inserts (Etymotic Research, IL, USA). Head localization coils were placed over the nasion and preauricular points to continuously monitor head movement.

#### Anatomical MRI acquisition and co-registration

The MEG head localization coils were replaced with multimodal radiographic markers, before acquiring structural MRI images; fiducial marking facilitated accurate co-registration of MEG and structural MRI data. The anatomical MRI scans were acquired at 3.0T on a Philips Achieva or Ingenia Elition scanner (Philips Medical Systems, International). Whole-brain 3D T1-weighted images were acquired using an MDEFT scan (flip angle = 90°, TE = 3.7 ms, TR = 8.1 ms, voxel size = 1.0 × 1.0 × 1.0 mm).

### Data analysis

#### Pre-processing and time-locked analysis of MEG

MEG data were processed using FieldTrip^40^ routines running in MATLAB version 2019b (Mathworks Inc., MA, USA). Line noise (60 Hz and harmonics at 120 Hz, 180 Hz) was attenuated using a very sharp discrete Fourier transform filter; the data were then bandpass filtered from 13 to 23 Hz, in order to isolate the signal (low-beta) of interest. Data were then epoched for both verb generation and control trials to 700–1200 ms post-stimulus onset. Epoched trials were then de-meaned and SQUID jump artifacts were automatically identified; trials containing artifact (range 0–4; *mean* ± *S.D.* = 1.68 ± 1.16) were rejected.

#### Head and source modelling

Individual 3D-T1 weighted images were segmented into brain, skull, and scalp compartments using SPM12 routines (FIL Methods Group, 2014). Realistic single-shell head models were constructed from the brain surface^41^.

A template source model, consisting of dipole positions placed at a regular, 10 mm interval inside the MNI152 brain, was developed for this study. The template positions were nonlinearly warped to each individual subject’s MRI, guaranteeing that all subjects had identical coverage for source analyses. Following source analyses in subject space, the individual dipole positions were assigned the corresponding MNI coordinates, facilitating group analyses.

#### Differential beamformer analyses with bootstrapping derived thresholds

Covariance matrices were computed from concatenated verb generation and control trials, for each participant. The time-series of sources were estimated using a linearly constrained minimum variance beamformer (LCMV) with 1% regularization^42^. The regularization was typical for this type of data and allowed for correct smoothing^43^. T-statistics were computed between the noun and noise conditions for low-beta power at each dipole position; the significance of each t-statistic was assessed through permutation testing (Monte Carlo simulation, as implemented in FieldTrip; condition labels randomly shuffled, 10,000 permutations, alpha of 0.05)^44^. ERD were defined as negative t-values to represent decreased spectral power, while ERS were defined as positive t-values to represent increased power.

#### Laterality index

A conventional laterality index (LI) was computed for individual participants from the total number of left (ERD_L_) versus right (ERD_R_) suprathreshold voxels, using the following formula:

$${LI}_{ERD}= \frac{\left({ERD}_{L}-{ERD}_{R}\right)}{\left({ERD}_{L}+{ERD}_{R}\right)}.$$

LI for ERS was also calculated using the same approach. These resulted in ERD and ERS LI scores ranging from − 1 to + 1 for each participant; − 1 represents completely right lateralized signal and + 1 represents completely left lateralized signal. LI scores of around 0 indicate approximately bilateral contribution.

Lateralization of ERD and ERS across development was assessed using Spearman’s rank correlation. To characterize potentially nonlinear developmental trajectories, we fit 1st through 3rd order polynomial functions to the data; the fits were assessed using Akaike’s Information Criteria (AIC). A corrected version—AIC_C_—which penalizes higher-order models inversely proportional to the sample size, was preferred.

Relationships between LI to handedness (EHI) was assessed by Spearman’s rank correlations; the relationship between LI and sex was assessed using independent samples t-tests.

## Results

### Whole brain spatial distribution of low-beta oscillations

Four participants were excluded due to a lack of significant low-beta ERD; as such, we could not be certain of their participation in the study paradigm. The final data pool consisted of 78 participants (42 females, ages 4.0 to 18.9 years). To facilitate comparison with previous studies, whole brain ERD/ERS was interpolated onto an MNI template brain then visualized as three groups consisting of the lower age tercile (Children, 4.0 to 7.6 years old, *n* = 26), middle tercile (Older Children, 7.6 to 13.3, *n* = 26) and upper age tercile (Adolescents, 13.4 to 18.9 years old, *n* = 26). Top 5% of voxels per tercile group average were visualized on standardized brain renderings in MRIcroGL (RRID: SCR_002403)^45^.

**Figure 1:**
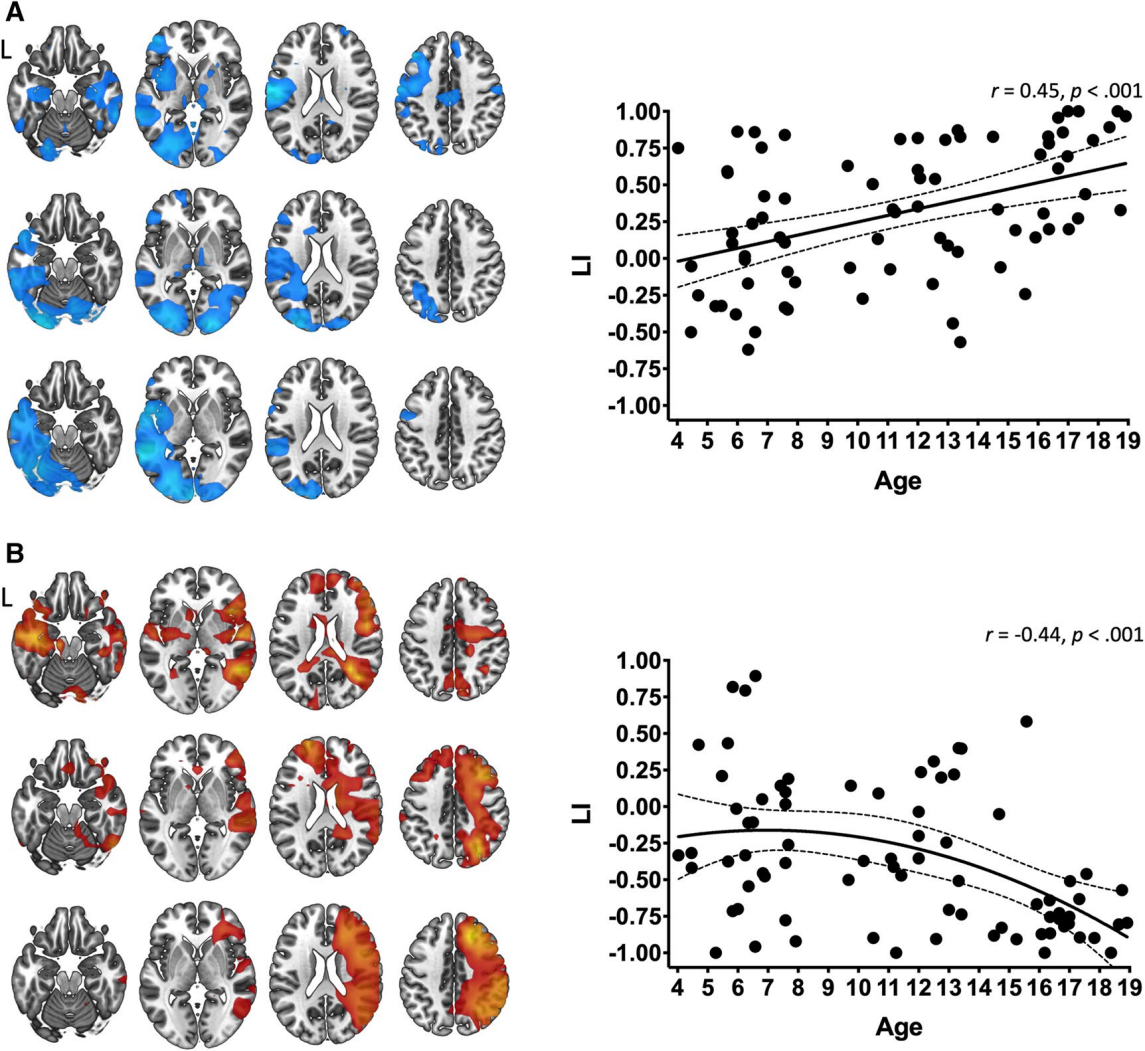
Whole-brain distribution of surviving neuromagnetic low-beta ERD (cool colors) and ERS (warm colors) during covert verb generation, and their corresponding laterality indices. The maps show top 5% of ERD or ERS for each age tercile. Within each contrast, the colour scaling is set from 95th to 100th percentiles. Across ages and contrasts, magnitude of t-values corresponding to percentiles are not consistent. Maps in (**A**) shows that children (above) elicit bilateral, diffuse and distributed low-beta ERD (above), which becomes less bilateral and more focalized for older children (middle) and is lateralized to the left perisylvian region in adolescents (below). The scatterplot of ERD LI (right) shows that ERD becomes increasingly left lateralized with age; the trajectory is linear across development. Maps in (**B**) show that ERS in children (above) is also bilateral but less so in older children (middle) and in adolescents (below) is lateralized to the right hemisphere. The scatterplot of ERS LI (right) shows that ERS becomes increasingly right lateralized with age; the trajectory is nonlinear (quadratic fit).

Whole brain ERD and ERS were distributed bilaterally and diffusely in the children group (Fig. 1A). In the adolescent group, we observed predominately left perisylvian ERD and right perisylvian ERS (Fig. 1B).

No relationship between handedness and ERD or ERS lateralization was found (*p* > 0.05). Nor did males and females differ in terms of lateralization or ERD or ERS (*p* > 0.05). Further, no relationship between estimates of verbal intelligence and ERD or ERS lateralization was found (*p* > 0.05).

AIC_C_ showed that ERD developmental trajectory was best fit with a linear model, while ERS was best fit with a quadradic curve (see Table 1). LIs of ERD and ERS were significantly negatively correlated (*r* =  − 0.49, *p* < 0.001). We observed a significant positive correlation between LI and age for ERD (*r* = 0.45, *p* < 0.001) (Fig. 1A) and a significant negative correlation between LI and age for ERS (*r* =  − 0.44, *p* < 0.001) (Fig. 1B). As reported previously, ERD was increasingly left lateralized with age. In contrast, ERS became increasingly right lateralized with age.

**Table 1:** Table of curve fit diagnostics for whole brain beta oscillation laterality in development.

Perturbation	Model	R^2^	adj. R^2^	AIC_c_
ERD	**Linear**	**0.20**	**0.19**	** − 134.2**
	Quadratic	0.21	0.21	** − **133.2
	Cubic	0.22	0.18	** − **131.5
ERS	Linear	0.18	0.17	** − **120.2
	**Quadratic**	**0.20**	**0.18**	** − 120.6**
	Cubic	0.20	0.17	− 118.4

Curve models are of laterality indices of low-beta event-related desynchronization and event-related synchronization as a function of age (n = 78).

Parsimonious model fit based on corrected Akaike’s Information Criterion (AIC_C_) is bolded.

### Frontal, parietal, and temporal lateralization

The covert verb generation task is known to elicit activity in the posterior brain, in regions supporting visual processing (often considered extra-linguistic). These potentially extra-linguistic activations affect the calculation of lateralization; as such, we developed a broad mask to assess oscillatory changes in frontal, parietal, and temporal lobes, only.

In children, ERD was diffuse and found bilaterally in the Inferior Frontal Gyrus (IFG) at *pars opercularis* and the left *pars triangularis* and *pars orbitalis*. It was also found bilaterally in the Inferior Frontal Sulcus (IFS) at the *ascending ramus* and left hemisphere *horizontal ramus*. ERD in children was also found bilaterally in the Medial Frontal Gyrus (MFG) and right hemisphere superior frontal gyrus (SFG). ERD in children was also found in the left posterior superior temporal gyrus (STG), left hemisphere medial temporal gyrus (MTG) and bilaterally in the Inferior Temporal Gyri (ITG). ERD among children was also found bilaterally in the insular cortex and temporal plane. Low-beta ERS was also more bilateral and diffuse in children than adolescents. ERS was found in the right IFG at *pars opercularis* and *pars orbitalis*. It was also found in the left temporal pole, and bilaterally in the MTG, medial temporal sulcus and insular cortex. Right hemispheric ERS in children was also found in the superior parietal lobule (SPL) and inferior parietal lobule (IPL) at the supramarginal gyrus.

For the adolescents, low beta ERD was found in almost exclusively in the left perisylvian region, specifically at the left IFG, left posterior STG and left insular cortex. However, low-beta ERS was almost exclusively in the right hemisphere. Sites of adolescent ERS included the IFG, IFS, MFG and sulcus, posterior STG, SPL, IPL at supramarginal and angular gyrus, and interparietal sulcus (Fig. S2).

**Figure 2:**
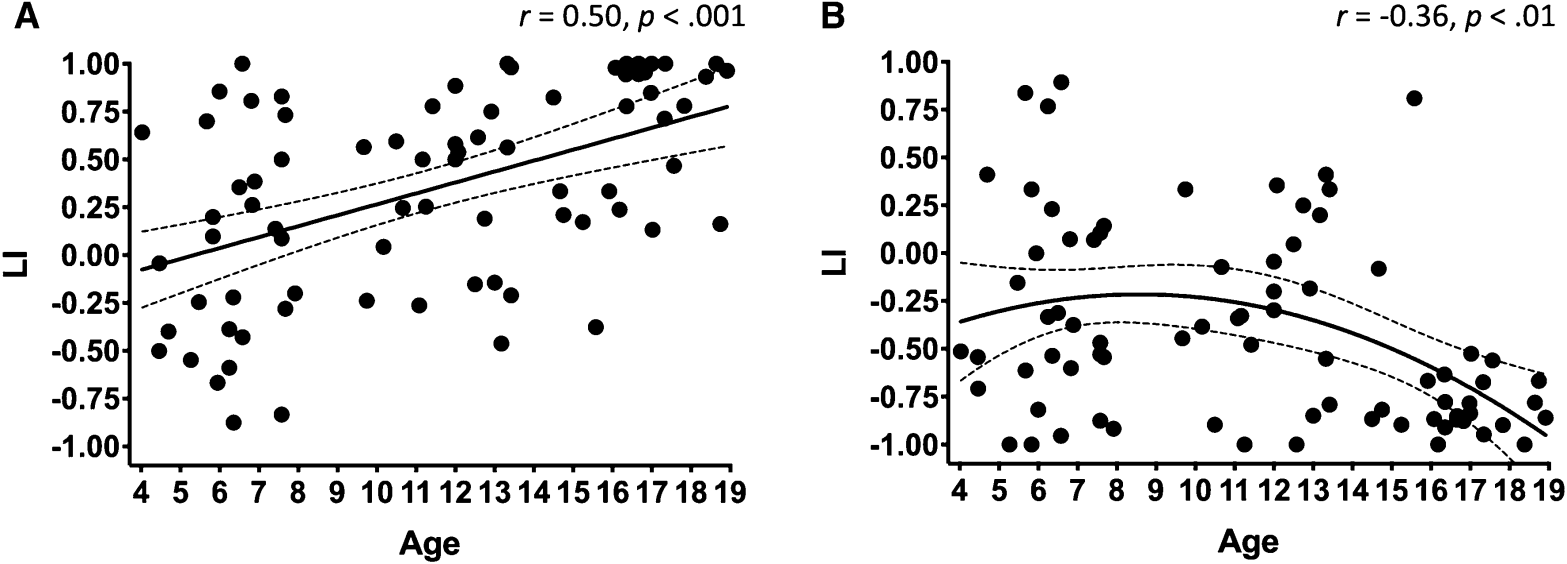
Frontal, temporal and parietal lobe laterality indices as a function of age. In (**A**), similar to the whole-brain findings, ERD becomes increasingly left lateralized with age with a linear trajectory across childhood. In (**B**), ERS becomes increasingly right lateralized with age with a nonlinear trajectory (quadratic fit).

Consist with the whole brain level, extra-occipital ERD developmental trajectory was best fit linearly, while ERS was best with a quadratic curve (see Table 2). LIs were taken for ERD and ERS in these extra-occipital cortices. The correlation between ERD and ERS LIs was significant (*r* =  − 0.43, *p* < 0.001). The correlation between age and LI was significant for ERD (*r* = 0.50, *p* < 0.001) and ERS (*r* =  − 0.36, *p* = 0.0012). Table 2 contains results of ERD and ERS LI curve fits, which are displayed in Fig. 2A,B, respectively.

**Table 2:** Table of polynomial fit diagnostics for extra-occipital (perisylvian lobes) beta oscillation laterality in development.

Perturbation	Model	R^2^	adj. R^2^	AIC_c_
ERD	**Linear**	**0.24**	**0.23**	− **115.6**
	Quadratic	0.24	0.22	− 113.6
	Cubic	0.24	0.17	− 111.4
ERS	Linear	0.12	0.11	− 109.6
	**Quadratic**	**0.17**	**0.15**	− **111.6**
	Cubic	0.17	0.13	− 109.3

Models are of laterality indices of low-beta event-related desynchronization and event-related synchronization as a function of age for each participant (n = 78).

Parsimonious model fit based on corrected Akaike’s Information Criterion (AIC_C_) is bolded.

## Discussion

This study provides novel information about the neuromagnetic correlates of expressive language. We assessed low-beta ERS in a cross-section of children performing covert verb generation in MEG and observed increasing right hemisphere lateralization with age. Variance in ERS lateralization also appeared to decrease with age. We suggest that the rightward lateralization of beta ERS reflects maturation of the language network in a normal developmental trajectory coupled with decreased plastic potential and possibly inhibition of superfluous neural activity that is not critical for language.

This right hemispheric ERS was contralateral to ERD, and the extent of lateralization for ERD and ERS was inversely related. In general, ERD and ERS lateralization are negatively correlated, but the fact that they follow distinct trajectories suggests that they are distinct epiphenomena, representing different processes. ERS peaks were found in the right hemisphere homologues of the canonical language network (i.e., Broca’s and Wernicke’s areas) and regions associated to speech-motor representations in children and adolescents. Adolescent ERS was also found in regions superior to the homologous perisylvian language areas on the right hemisphere. We argue that emergent right hemisphere ERS indexes *neural commitment*, suggesting a loss of potential for supporting language, in the context of a neurological insult^46^. Further, we argue that age-related lateralization of ERS, may provide a convenient demarcation for decreased plastic potential as a function age.

Thus, lateralization and maturation in language appears to be successfully mapped by ERS but for regions that are contralateral to the canonical language network. Based on these results, we propose that right hemisphere ERS may assay plastic potential in the human brain and inform clinical practices that may produce a risk of impairments to language functions.

Previous investigators have suggested that beta ERS may be a signature of cortical idling and more recently, neuronal inhibition. This signature tends not to show neural activity as a BOLD-fMRI signal for language in patients or healthy controls^47,48^ but does indeed appear as a strong signal across macroscale regions of the right hemisphere. It is possible that the right hemisphere regions showing ERS may be actively inhibited as a result of being competitive to expressive language, allowing the processes of these regions to be disengaged or suppressed during language production while regions critically committed to language operate. Further, this active inhibition for language appears to be transient, emerging in a task-relevant fashion, which differentiates it from a resting state baseline of rhythms occurring during cognitive inactivity. If this is the case, language might emerge from a finely tuned transient balance of excitatory and inhibitory neuronal functioning^49^. Further, based on the healthy participant group of this study, deviation from this proposed normal developmental trajectory of synchronous oscillatory lateralization may associate to cortical lesions, neuropathology or neural disorders. For the current task, perhaps the excitatory functions are predominantly occurring in the canonical language network, but active inhibition may occur in the right hemisphere to achieve balanced allocation of neural resources. Importantly, if ERS indexes neuronal inhibition, the precise role of right hemisphere neurons during speech and language expression may be established from these results.

We note that limitations to the task and analyses here controlled for non-literal semantic processing, semantic distance, prosody, valence, orthographic processing and other language related phenomena that might involve contributions from the right hemisphere^50^. Further, socioeconomic factors that could explain variance in the early childhood years were not considered in these analyses. Finally, more quantitative approaches characterizing the trajectory may be useful in future, large-scale studies. To avoid overfitting, we refrain from further quantitation on the fitted trajectory.

In conclusion, our results suggest that development of the language network involves functional lateralization of neuronal processing that associate to both low-beta ERD and also ERS. We also suggest that the results found in this study reveal limits to plastic potential that are shown to be incrementally increased by ERD lateralization and abruptly increased by ERS lateralization during adolescence. This abrupt increase may result in perdurable loss of regions available for neuroplastic transfer of language function. This may be of relevance to clinical insult to the brain in procedures related to epilepsy, tumor removal and other conditions. Finally, low-beta ERS in the right hemisphere may lateralize across development for language, which could be indexing neuronal inhibition. Fundamentally, as language processing matures, regions that might be sensitized to language output properties respond differently across the brain, but these responses are nevertheless more focalized as the network develops, regardless of the direction of changes in oscillatory power.

## Supplementary Information

Supplementary Information.

## References

[1] Binder JR, Frost JA, Hammeke TA, Cox RW, Rao SM, Prieto T (1997). Human brain language areas identified by functional magnetic resonance imaging. J. Neurosci..

[2] Friederici AD (2011). The brain basis of language processing: From structure to function. Physiol. Rev..

[3] Friederici AD (2012). The cortical language circuit: From auditory perception to sentence comprehension. Trends Cogn. Sci..

[4] Gabrieli JD, Poldrack RA, Desmond JE (1998). The role of left prefrontal cortex in language and memory. Proc. Natl. Acad. Sci. U.S.A..

[5] Geschwind N (1970). The organization of language in the brain. Science.

[6] Hirata M, Kato A, Taniguchi M, Saitoh Y, Ninomiya H, Ihara A, Yoshimine T (2004). Determination of language dominance with synthetic aperture magnetometry: Comparison with the Wada test. NeuroImage.

[7] Kadis DS, Pang EW, Mills T, Taylor MJ, McAndrews MP, Smith ML (2011). Characterizing the normal developmental trajectory of expressive language lateralization using magnetoencephalography. J. Int. Neuropsychol. Soc..

[8] Lohmann G, Hoehl S, Brauer J, Danielmeier C, Bornkessel-Schlesewsky I, Bahlmann J, Friederici A (2010). Setting the frame: The human brain activates a basic low-frequency network for language processing. Cereb. Cortex.

[9] Price CJ (2000). The anatomy of language: Contributions from functional neuroimaging. J. Anat..

[10] Purves D, Augustine G, Fitzpatrick D, Katz L, LaMantia A-S, McNamara J, Williams M, Purves D, Augustine GJ, Fitzpatrick D, Katz LC, LaMantia AS, McNamara JO, Williams SM (2004). Language and lateralization. Neuroscience.

[11] Toga AW, Thompson PM (2003). Mapping brain asymmetry. Nat. Rev. Neurosci..

[12] Turken AU, Dronkers NF (2011). The neural architecture of the language comprehension network: Converging evidence from lesion and connectivity analyses. Front. Syst. Neurosci..

[13] Youssofzadeh V, Vannest J, Kadis DS (2018). fMRI connectivity of expressive language in young children and adolescents. Hum. Brain Mapp..

[14] Holland SK, Plante E, Byars AW, Strawsburg RH, Schmithorst VJ, Ball WS (2001). Normal fMRI brain activation patterns in children performing a verb generation task. NeuroImage.

[15] Holland SK, Vannest J, Mecoli M, Jacola LM, Tillema J, Karunanayaka PR, Schmithorst VJ, Yuan W, Plante E, Byars W (2007). Functional MRI of language lateralization during development in children. Int. J. Audiol..

[16] Kadis DS, Iida K, Kerr EN, Logan WJ, McAndrews MP, Ochi A, Otsbudo H, Rutka JT, Snead OC, Weiss SK, Smith ML (2007). Intrahemispheric reorganization of language in children with medically intractable epilepsy of the left hemisphere. J. Int. Neuropsychol. Soc..

[17] Rasmussen T, Milner B (1977). The role of early left-brain injury in determining lateralization of cerebral speech functions. Ann. N. Y. Acad. Sci..

[18] Szaflarski JP, Holland SK, Schmithorst VJ, Byars AW (2006). fMRI study of language lateralization in children and adults. Hum. Brain Mapp..

[19] Vannest J, Karunanayaka PR, Schmithorst VJ, Szaflarski JP, Holland SK (2009). Language networks in children: Evidence from functional MRI studies. Am. J. Roentgenol..

[20] Pfurtscheller G (1997). EEG event-related desynchronization (ERD) and synchronization (ERS). Electroencephalogr. Clin. Neurophysiol..

[21] Klimesch W, Doppelmayr M, Wimmer H, Gruber W, Röhm D, Schwaiger J, Hutzler F (2001). Alpha and beta band power changes in normal and dyslexic children. Clin. Neurophysiol..

[22] Neuper C, Wörtz M, Pfurtscheller G, Neuper C, Klimesch W (2006). ERD/ERS patterns reflecting sensorimotor activation and deactivation. Event-Related Dynamics of Brain Oscillations.

[23] Pfurtscheller G, Lopes da Silva FH (1999). Event-related EEG/MEG synchronization and desynchronization: Basic priniciples. Clin. Neurophysiol..

[24] Jensen O, Mazaheri A (2010). Shaping functional architecture by oscillatory alpha activity: Gating by inhibition. Front. Hum. Neurosci..

[25] Jensen O, Goel P, Kopell N, Pohja M, Hari R, Ermentrout B (2005). On the human sensorimotor-cortex beta rhythm: Sources and modeling. NeuroImage.

[26] Hall EL, Robson SE, Morris PG, Brookes MJ (2014). The relationship between MEG and fMRI. NeuroImage.

[27] Pang EW, Wang F, Malone M, Kadis DS, Donner EJ (2011). Localization of Broca’s area using verb generation tasks in the MEG: Validation against fMRI. Neurosci. Lett..

[28] Yuan H, Liu T, Szarkowski R, Rios C, Ashe J, He B (2010). Negative covariation between task-related responses in alpha/beta-band activity and BOLD in human sensorimotor cortex: An EEG and fMRI study of motor imagery and movements. NeuroImage.

[29] Bishop DV, Hardiman MJ, Barry JG (2010). Lower-frequency event-related desynchronization: A signature of late mismatch response to sounds, which is reduced or absent in children with specific language impairments. J. Neurosci..

[30] Foley E, Cross JH, Thai NJ, Walsh AR, Bill P, Furlong P, Wood AG, Cerquiglini A, Seri S (2019). MEG assessment of expressive language in children evaluated for epilepsy surgery. Brain Topogr..

[31] Findlay AM, Ambrose JB, Cahn-Weiner DA, Houde JF, Honma S, Hinkley LB, Berger MS, Nagarajan SS, Kirsch HE (2012). Dynamics of hemispheric dominance for language assessed by magnetoencephalographic imaging. Ann. Neurol..

[32] Hinkley L, De Witte E, Cahill-Thompson M, Mizuiri D, Garrett C, Honma S, Findlay A, Gorno-Tempini ML, Tarapore P, Kirsch HE, Mariën P, Houde JF, Berger M, Nagarajan SS (2020). Optimizing magnetoencephalographic imaging estimation of language lateralization for simpler language tasks. Front. Hum. Neurosci..

[33] Traut T, Sardesh N, Bulubas L, Findlay A, Honma SM, Mizuiri D, Berger MS, Hinkley LB, Nagarajan SS, Tarapore PE (2018). MEG imaging of recurrent gliomas reveals functional plasticity of hemispheric language specialization. Hum. Brain Mapp..

[34] Lukic S, Barbieri E, Wang X, Caplan D, Swathi K, Rapp B, Parrish TB, Thompson CK (2017). Right hemisphere grey matter volume and language functions in stroke aphasia. Neural Plast..

[35] Meltzer JA, Suraji W, Ryder J, Solomon B, Braun AR (2013). Adaptive significance of right hemisphere activation in aphasic language comprehension. Neuropsychologia.

[36] Oldfield RC (1971). The assessment and analysis of handedness: The Edinburgh inventory. Neuropsychologia.

[37] Dunn LM, Dunn DM, Lenhard A (2015). Peabody Picture Vocabulary Test: PPVT 4.

[38] Williams K (2007). Expressive Vocabulary Test.

[39] Semel EM, Wiig EH, Secord W (2004). CELF 4: Clinical Evaluation of Language Fundamentals.

[40] Oostenveld R, Fries P, Maris E, Schoffelen JM (2011). FieldTrip: Open source software for advanced analysis of MEG, EEG, and invasive electrophysiological data. Comput. Intell. Neurosci..

[41] Nolte G (2003). The magnetic lead field theorem in the quasi-static approximation and its use for magnetoencephalography forward calculation in realistic volume conductors. Phys. Med. Biol..

[42] Van Veen BD, van Drongelen W, Yuchtman M, Suzuki A (1997). Localization of brain electrical activity via linearly constrained minimum variance spatial filtering. IEEE Trans. Biomed. Eng..

[43] Brookes MJ, Vrba J, Robinson SE, Stevenson CM, Peters AM, Barnes GR, Hillebrand A, Morris PG (2008). Optimising experimental design for MEG beamformer imaging. NeuroImage.

[44] Maris E, Oostenveld R (2007). Nonparametric statistical testing of EEG- and MEG-data. J. Neurosci. Methods.

[45] Rorden C, Brett M (2000). Stereotaxic display of brain lesions. Behav. Neurol..

[46] Devlin AM, Cross JH, Harkness W, Chong WK, Harding B, Vargha-Khadem F, Neville BGR (2003). Clinical outcomes of hemispherectomy for epilepsy in childhood and adolescence. Brain.

[47] Hamberger MJ, Cole J (2011). Language organization and reorganization in epilepsy. Neuropsychol. Rev..

[48] Tivarus ME, Starling SJ, Elissa EL, Langfitt JT (2012). Homotopic language re-organization in the right hemisphere after early left hemisphere injury. Brain Lang..

[49] Ponzi A, Wickens J (2010). Sequentially switching cell assemblies in random inhibitory networks of spiking neurons in the striatum. J. Neurosci..

[50] Lindell AK (2006). In your right mind: Right hemisphere contributions to language processing and production. Neuropsychol. Rev..

