# Supplementary material for: Beta synchrony for expressive language lateralizes to right hemisphere in development
Source: Sci Rep. 2021 Feb 17;11:3949. doi: 10.1038/s41598-021-83373-z (PMC7889886; doi:10.1038/s41598-021-83373-z)
Supplement: Supplementary file 1 — Supplementary Information. [file 41598_2021_83373_MOESM1_ESM.docx]

Beta synchrony for expressive language lateralizes to right hemisphere in development: Supplementary Images

Vivek V. Sharma^1^, Jennifer Vannest^2, 3^, Hansel M. Greiner^4^,

Hisako Fujiwara^4^, Jeffrey R. Tenney^4^, Brady J. Williamson^5^, *Darren S. Kadis^1, 6^

^1^ Neurosciences and Mental Health, Hospital for Sick Children, Toronto, ON, Canada

^2^ Communication Sciences & Disorders, University of Cincinnati, OH, USA

^3^ Division of Speech-Language Pathology, Cincinnati Children’s Hospital Medical Center, OH

^4^ Division of Neurology, Cincinnati Children’s Hospital Medical Center, OH

^5^ Department of Radiology, University of Cincinnati, OH, USA

^6^ Department of Physiology, Faculty of Medicine, University of Toronto, ON

*Correspondence should be addressed to:

Dr. Darren S. Kadis

Neurosciences and Mental Health

Hospital for Sick Children

686 Bay Street, Toronto ON M5G 0A4

darren.kadis@sickkids.ca


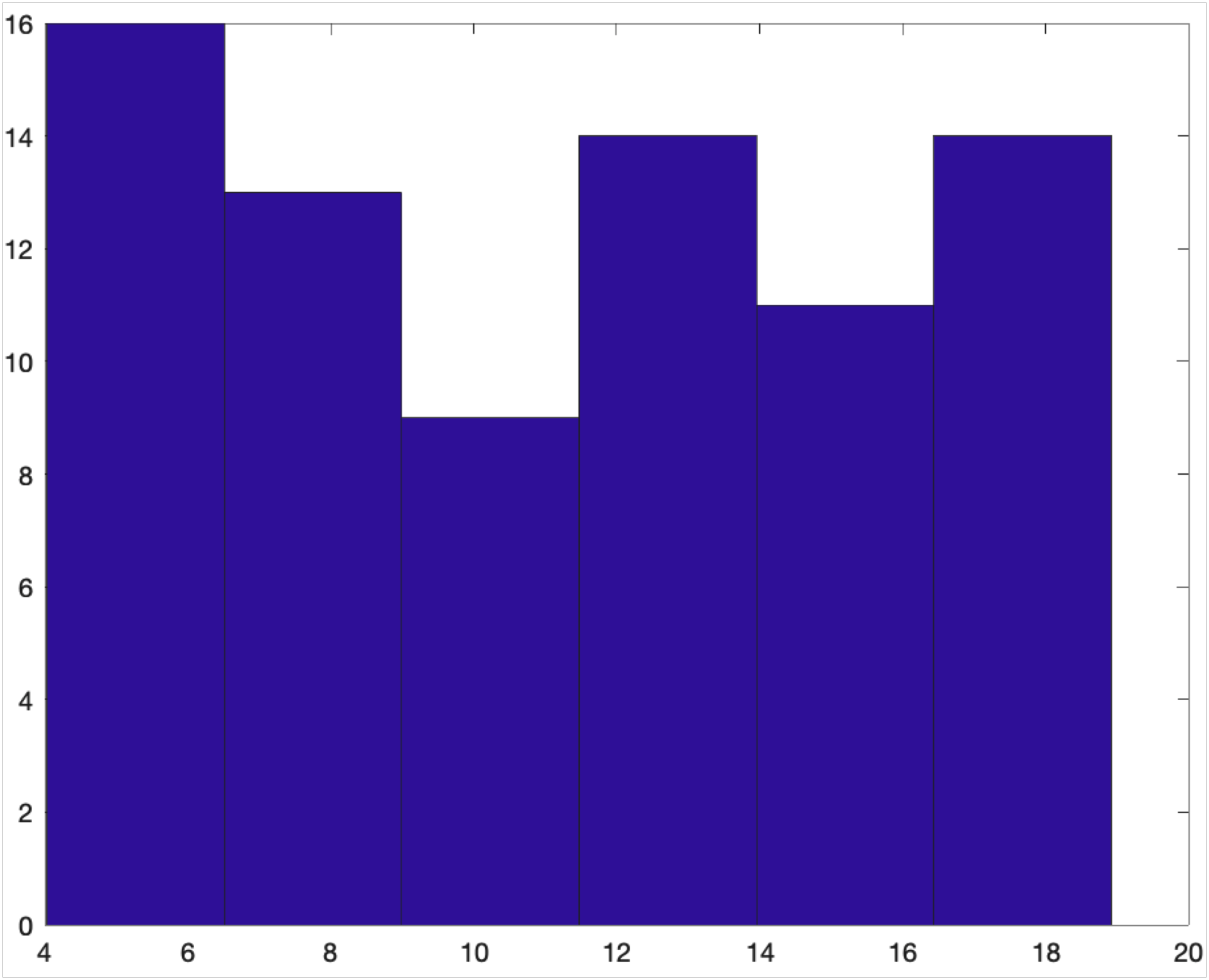


**Supplementary Figure 1.** The age distribution of participants in six bins. The ages range from ages 4.0 to 19.0 years old. In increasing order, the bins are: 1. *n* = 16 (center = 5.71, range = 4.0 to 6.5); 2. *n* = 13 (center = 7.8, range = 6.5 to 9.0); 3. *n* = 9 (center = 10, range = 9.0 to 11.5); 4. *n* = 14 (center = 12.7, range = 11.5 to 14.0); 5. *n* = 11 (center = 15.2, range = 14.0 to 16.4); 6. *n* = 14 (center = 17.7, range = 16.4 to 19.0).


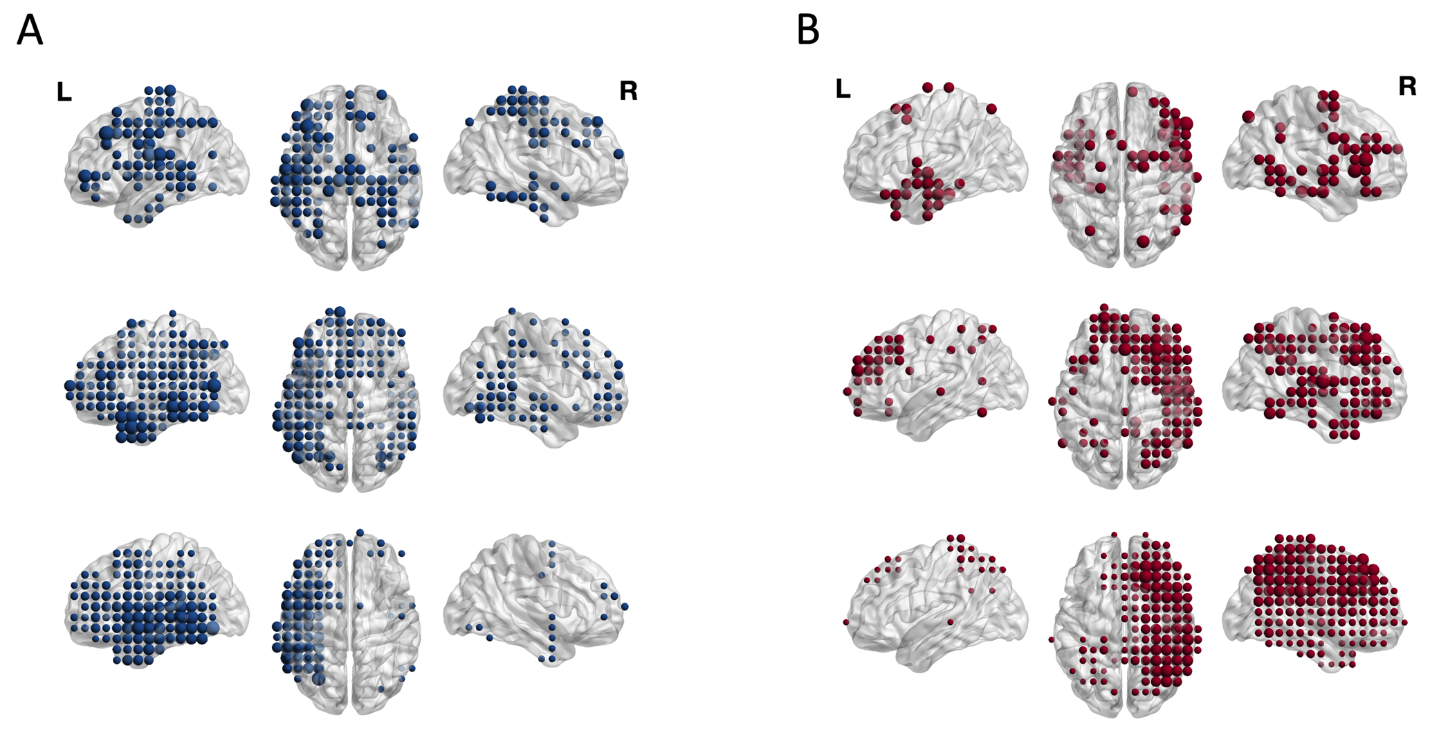


**Supplementary Figure 2.** Beamformed neuromagnetic nodes of event-related desynchronization (ERD) in blue and event-related synchronization (ERS) in red. Each figure shows left lateral, superior, and right lateral views of the cerebral surface, from left to right. Nodes are the mean of binary values representing significant ERD and ERS for non-occipital voxels, averaged across participants within the age tercile groups. In (A), ERD of Tercile 1 (above), Tercile 2 (middle), and Tercile 3 (below) show bilateral to left lateralized responses across development when generating verbs related to a spoken noun. However, (B) shows group mean ERD moving from bilateral to right lateralized responses across development.
